# Climate Change and American Bullfrog Invasion: What Could We Expect in South America?

**DOI:** 10.1371/journal.pone.0025718

**Published:** 2011-10-03

**Authors:** Javier Nori, J. Nicolás Urbina-Cardona, Rafael D. Loyola, Julián N. Lescano, Gerardo C. Leynaud

**Affiliations:** 1 Centro de Zoología Aplicada, Facultad de Ciencias Exactas, Físicas y Naturales, Universidad Nacional de Córdoba, Córdoba, Argentina; 2 Departamento de Ecología y Territorio, Facultad de Estudios Ambientales y Rurales, Pontificia Universidad Javeriana, Bogota, Colombia; 3 Departamento de Ecologia, Universidade Federal de Goiás, Goiás, Brazil; 4 CONICET, Buenos Aires, Argentina; Macquarie University, Australia

## Abstract

**Background:**

Biological invasion and climate change pose challenges to biodiversity conservation in the 21^st^ century. Invasive species modify ecosystem structure and functioning and climatic changes are likely to produce invasive species' range shifts pushing some populations into protected areas. The American Bullfrog (*Lithobates catesbeianus*) is one of the hundred worst invasive species in the world. Native from the southeast of USA, it has colonized more than 75% of South America where it has been reported as a highly effective predator, competitor and vector of amphibian diseases.

**Methodology/Principal Findings:**

We modeled the potential distribution of the bullfrog in its native range based on different climate models and green-house gases emission scenarios, and projected the results onto South America for the years of 2050 and 2080. We also overlaid projected models onto the South American network of protected areas. Our results indicate a slight decrease in potential suitable area for bullfrog invasion, although protected areas will become more climatically suitable. Therefore, invasion of these sites is forecasted.

**Conclusion/Significance:**

We provide new evidence supporting the vulnerability of the Atlantic Forest Biodiversity Hotspot to bullfrog invasion and call attention to optimal future climatic conditions of the Andean-Patagonian forest, eastern Paraguay, and northwestern Bolivia, where invasive populations have not been found yet. We recommend several management and policy strategies to control bullfrog invasion and argue that these would be possible if based on appropriate articulation among government agencies, NGOs, research institutions and civil society.

## Introduction

Climate changes are likely to affect the distributional ranges of invasive species [1]–[4], which are one of the most serious global threats for biodiversity [5], [1]. Invasive species can modify ecosystem processes and affect ecosystem structure and functioning [6]–[9], with economic impacts reaching billions of dollars [10]. Worldwide, many invasive species have colonized protected areas altering their ecological integrity [7], [11]. However, management actions established within protected areas or along buffer zones that try to control invasive species are usually ineffective given that many threats come from outside the area itself [12].

The American Bullfrog (*Lithobates catesbeianus*) is endemic to eastern North America and has been introduced in approximately 40 countries in four continents via aquaculture and the aquarium trade [13]. It has been considered one of the hundred worst invasive species in the world [14]. The negative effects of the American Bullfrog invasion on native species arise from competition, amphibian and fish predation, as well as the spread of ranavirus and the fungus *Batrachochytrium dendrobatidis,* which is systematically killing amphibians in pristine environments and protected areas [15], [16]. Specifically in South America, *L. catesbeianus* has been reported in ten countries [17]–[19].

In recent years, species distribution models (SDMs) have been widely used to predict ecologically suitable areas for the establishment of invasive species under current and future climate projections with the goal of pinpointing regions in which urgent preventive actions must be taken (see Franklin [20] for a comprehensive revision of SDM theory and applications). SDMs combine presence data of individuals within their known distribution range with climatic data from those same areas to generate models usually describing the Grinnelian niche of the organism [21], estimate their current distribution and predict areas exhibiting the same or similar environmental space.

Several authors have already developed predictive models for the American Bullfrog distribution across the globe or South America. Ficetolla et al. [22] proposed a global potential distribution (at current conditions) mostly aimed at predicting the potential distribution of *L. catesbeianus* in Europe. Giovanelli et al. [23] and Nori et al. [24] modeled potential distributions of the species in Brazil and Argentina, respectively (at current conditions), concluding that the presence of this species in the Atlantic Forest Biodiversity Hotspot is of special concern in the continent. Urbina-Cardona and Castro [25] modeled bullfrog distribution in Colombia at present as well as in a future scenario (2050) and determined that the species tends to slightly reduce its suitable range in the future. However, these results contrast with the climate change models proposed by Urbina-Cardona et al. [26] which identify vulnerable areas of massive future expansion in the Caribbean, Amazon and Orinoquia regions. Finally, Loyola et al. [27] evaluated the impact of a *L. catesbeianus* invasion in the Brazilian Atlantic Forest protected areas using ensembles of forecasts based on different modeling algorithms and future climatic models. They suggest that protected areas are more likely to be invaded by the species in the future due to the climatic changes expected for the region.

Here, we modeled the potential distribution of *L. catesbeianus* in its native range based on different climatic models and projected the result onto all of continental South America under different time slices (present time, 2050, and 2080). We then overlapped all of the projected models onto the IUCN layers of terrestrial protected areas. Our main goals were to determine: (a) the potential distribution of the American Bullfrog in South America applying recent suggested approaches for modeling invasive species ranges, (b) the pattern of change in the potential suitable habitats of *L. catesbeianus* during different time slices of climate change scenarios, (c) changes in the potential suitable surface of *L. catesbeianus* under different climatic models during the same time period, and (d) the surface of environmentally suitable IUCN protected areas for *L. catesbeianus* in different time slices and under different climate models. Lastly, we compared our results with previous related research.

## Materials and Methods

### Study area and species occurrence data

We focused our analyses in all of South America (Argentina, Bolivia, Brazil, Chile, Colombia, Ecuador, French Guiana, Guyana, Paraguay, Peru, Suriname, Uruguay and Venezuela) spanning a total area of 17.825.184 km^2^.

We began our study with a dataset of 1431 individual records from the native range of *L. catesbeianus*, obtained from HerpNet (http://www.herpnet.org), CONABIO (http://www.conabio.gob.mx/remib/doctos/remib_esp.html) and GBIF (http://data.gbif.org), including occurrences in Mexico, USA and Canada (Fig. S1). Additionally, we used 210 individual records of the American Bullfrog in South America, obtained from I3N IABIN (http://i3n.iabin.net), Species Link (http://splink.cria.org.br), herpetological collections (Instituto Hórus, Universidad de Antioquia, Centro de Zoología Aplicada of the Universidad Nacional de Córdoba), and relevant literature [22], [23], [24], [28], [29] and from the “spatial download data” section of the IUCN Red List of Threatened Species web site [30]. Georeferencing was conducted when necessary using the Alexandria Digital Library Gazetteer (http://middleware.alexandria.ucsb.edu/client/gaz/adl/index.jsp). Duplicate records were discarded via ENMTools 1.3 [31].

### Climatic data

We did a pairwise Pearson correlation between 19 bioclimatic and one topographic variable at a spatial resolution of 30 seconds. We selected ten variables that did not showed colinearity with other variables (r<0.75): Mean Diurnal Range, Isothermality, Maximum Temperature of Warmest Month, Temperature Annual Range, Mean Temperature of Wettest Quarter, Mean Temperature of Warmest Quarter, Precipitation of Wettest Month, Precipitation Seasonality, Precipitation of Driest Quarter and Altitude (Table S1). To estimate the influence of global climate change on the potential distribution of *L. catesbeianus*, we modeled the distribution of the species for three different time slices: present, 2050, and 2080. Due to the large effect of different Atmosphere-Ocean Global Circulation Models (AOGCMs) in species range projections [32], [33], we selected three different AOGCMs (CCCMA-CGCM31, CSIRO_MK30 and IPSL_CM4) for each time slice. The selected AOGCMs for this research, are widely used in the literature, additionally they have different equilibrium climate sensitivity values ranging from 3.1°C to 4.4°C. Equilibrium climate sensitivity is the annual mean surface air temperature change experienced by the climate system after it has attained a new equilibrium in response to a doubling of CO_2_ concentration, and are within the range of all AOGCMs available from IPCC [34]. We compiled current and future climatic data from the Worldclim database (www.worldclim.com) [35]. Future scenarios were developed by IPCC's Fourth Assessment Report (AR4). All climatic layers were clipped to (a) 13 countries of continental South America, and (b) the native range of *L. catesbeianus* (Fig. S1).

### Modeling Method

We modeled the potential distribution of *L. catesbeianus* in its native range. We separated the 1431 individual records into two groups, one for training the models (1074 records) and one for testing them (357 records). The resulting models were projected for all of South America, in both current and potential future environments for the three different AOGCMs. We used MaxEnt 3.3.3e [36] since it has been shown to be a robust method for presence-only datasets [37], [38]. We ran the MaxEnt models using the default setting, except for when selecting regularization values. This parameter was determined by the application of the small sample corrected variant of Akaike's Information Criterion (AIC) implemented in ENMTools 1.3 (for details see [31]).

The resulting outputs of MaxEnt were continuous maps, which allowed us to make fine distinctions between the modeled suitability of different areas. A “minimum training presence” value was used to discriminate suitable from non-suitable habitat, which minimized both the training and test omission rates without resulting in an overly general model. This value has been proposed in recent papers for modeling the range of invasive species (e.g. [38], [39]). We assessed model performance using 25% of the records as “test data” in order to calculate the area under the receiver operating characteristic curve (AUC/ROC) [40].

We projected the resulting models of the species' native range, defined as a minimum convex polygon (Fig. S1). To avoid spurious projections (or to search for novel climate conditions), we used the “clamping analysis” (implemented in MaxEnt). This analysis treats variables outside the training range as if they were at the limit of the training range. This allows the identification of locations where predictions are uncertain because of the method of extrapolation by showing where clamping substantially affects the predicted value [36], [41]. We validated our results by plotting the actual reported populations for South America onto the predicted distribution map (for present conditions). Finally, we evaluated the similarity between the modeled results for different AOGCMs during the same period by applying *I* statics and Relative Rank (RR) with the latest version of ENMTools [31].

### Protected areas analysis

The shape files of the protected areas of continental South America were obtained from the World Database of Protected Areas (http://protectedplanet.net/). We only considered “designated” protected areas in any of the six IUCN categories. We overlapped the resulting models onto the current network of South American protected areas to determine the potential surface of protected areas that are environmentally suitable for *L. catesbeianus*. We also did this for different time slices and AOGCMs. Finally, we also calculated the surfaces of the different IUCN categories using ArcGIS 9.3.1 [42].

## Results

The predictive models had high AUC values (0.842+/−0.009 for test and 0.86+/−0.011 for training). The “minimum training presence” value was low (0.094) and the better regularization value (lower value of AICc) was 1. Both the *I* statics and the Relative Rank (RR) [31] reflected the highest values of similarity between results of different AOGCMs for the same time slice. The *I* statics index varied from 0.88 to 0.91 in the 2050 results and from 0.79 to 0.86 in the 2080 results. The RR varied between 0.84 and 0.91 (2050) and between 0.79 and 0.85 (2080).

**Figure 1:**
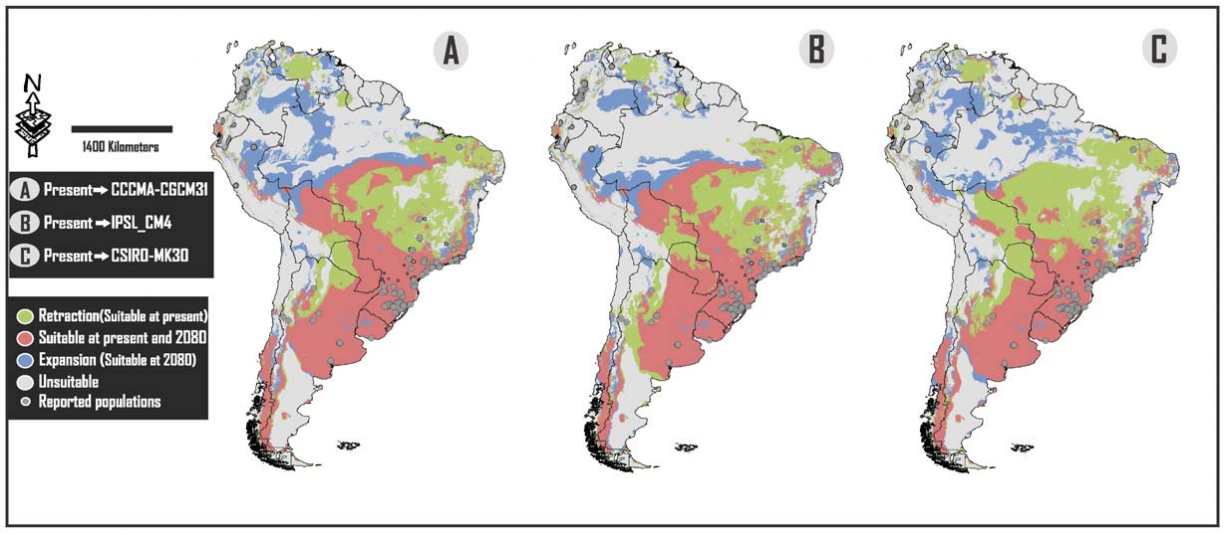
Comparison between results of projections at present and 2080. Each map shows potential suitable areas for *Lithobates catesbeianus* at one of the three different analyzed AOGCMs, classified in: Retraction (suitable areas at present but not at 2080), Expansion (suitable areas at 2080 but not at present conditions) and suitable areas at present and at 2080.

**Figure 2:**
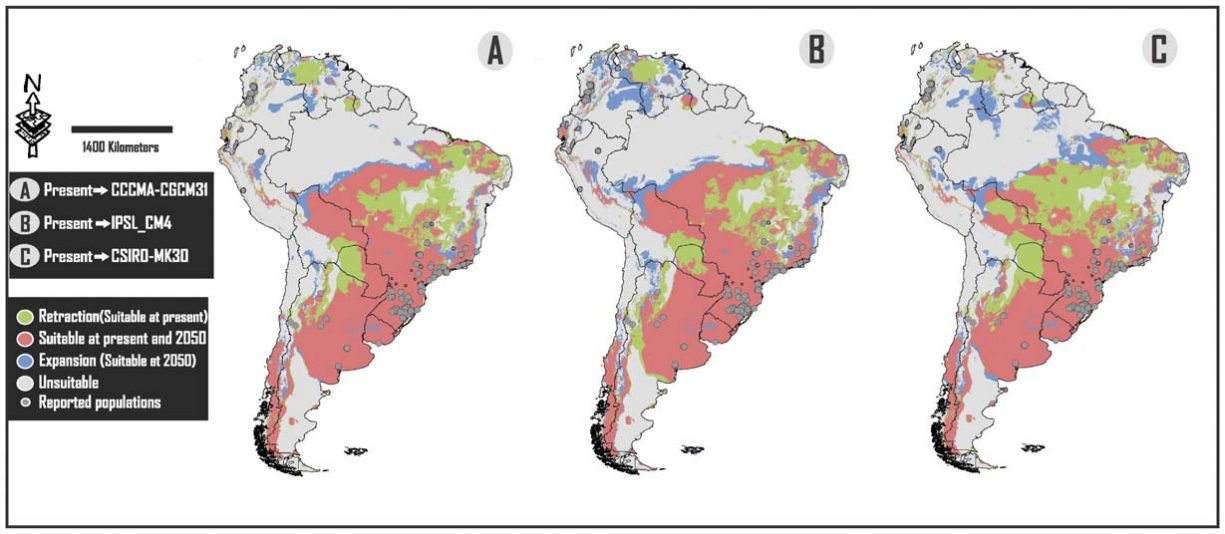
Comparison between results of projections at present and 2050. Each map shows potential suitable areas for *Lithobates catesbeianus* at one of the three different analyzed AOGCMs, classified in: Retraction (suitable areas at present but not at 2050), Expansion (suitable areas at 2050 but not at present conditions) and suitable areas at present and at 2050.

**Figure 3:**
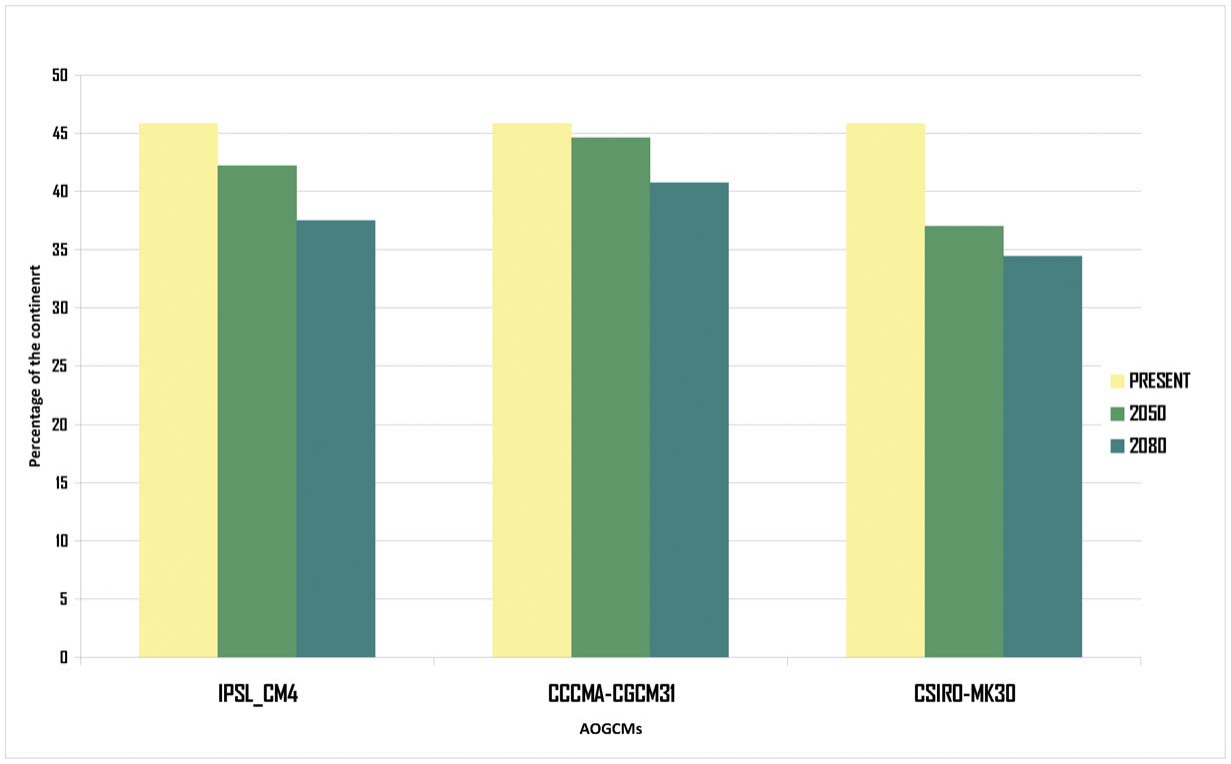
Histogram of suitable surface. Percentage of suitable surface of the entire continent for *Lithobates catesbeianus* at the three analyzed time slices and AOGCMs.

The geographic projection of the model in current conditions was in remarkable concordance with the reported feral populations of the American Bullfrog in South America. The results of the analyses reflected a slight decrease in the potential suitable areas for this invasive species in the future (Fig. 1, 2, 3). At current conditions, the species is predicted to be absent from a major portion of northwestern and central eastern parts of the continent as well as in the southeastern portion of Argentina. Additionally, in the future, the invasion could retract in central western Brazil as well as in a big portion of Argentina, Paraguay and Bolivia, but increase in northern Brazil, southeastern Colombia, eastern Peru and southern Venezuela (Fig. 3).

**Figure 4:**
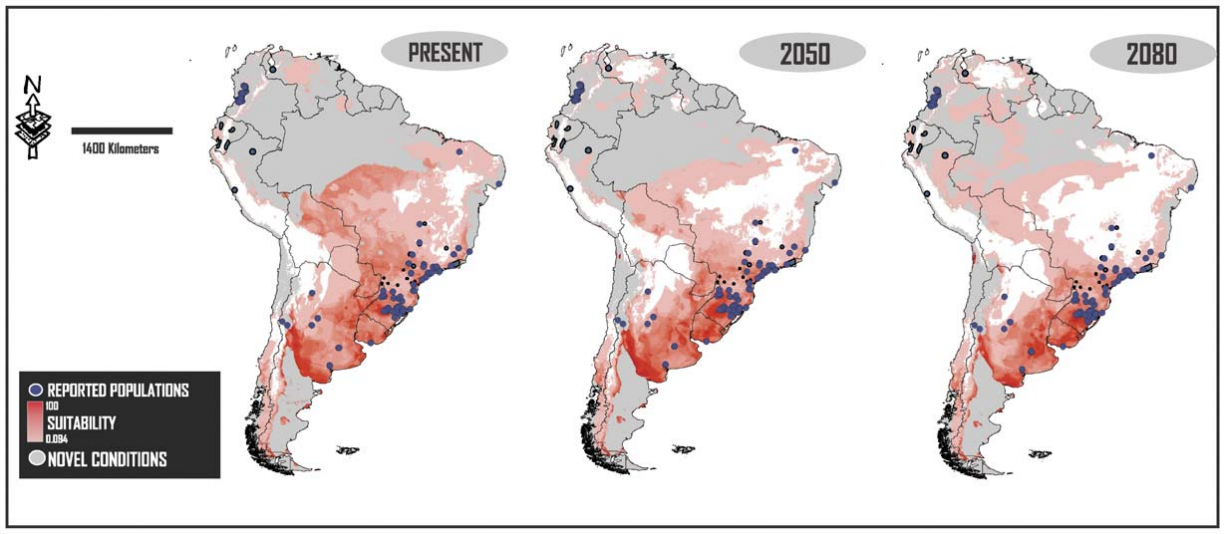
Suitability values and novels conditions of the CCCMA-CGCM31 scenario. Each map shows suitability values (red gradient) and novel conditions (gray) at one of the three analyzed time slices.

Areas with highest values of suitability for *L. catesbeianus* were located in the southern portions of Brazil and Uruguay, and in north and central eastern Argentina. Figure 4 shows the suitability values of one of the AOGMs scenarios (CCCMA_CGCM31) at all of the studied time slices, however, all scenarios showed the same suitability pattern. The geographic projections of future scenarios showed an increase in the suitability values of the southern portion of Brazil and Uruguay and central eastern Argentina.

**Figure 5:**
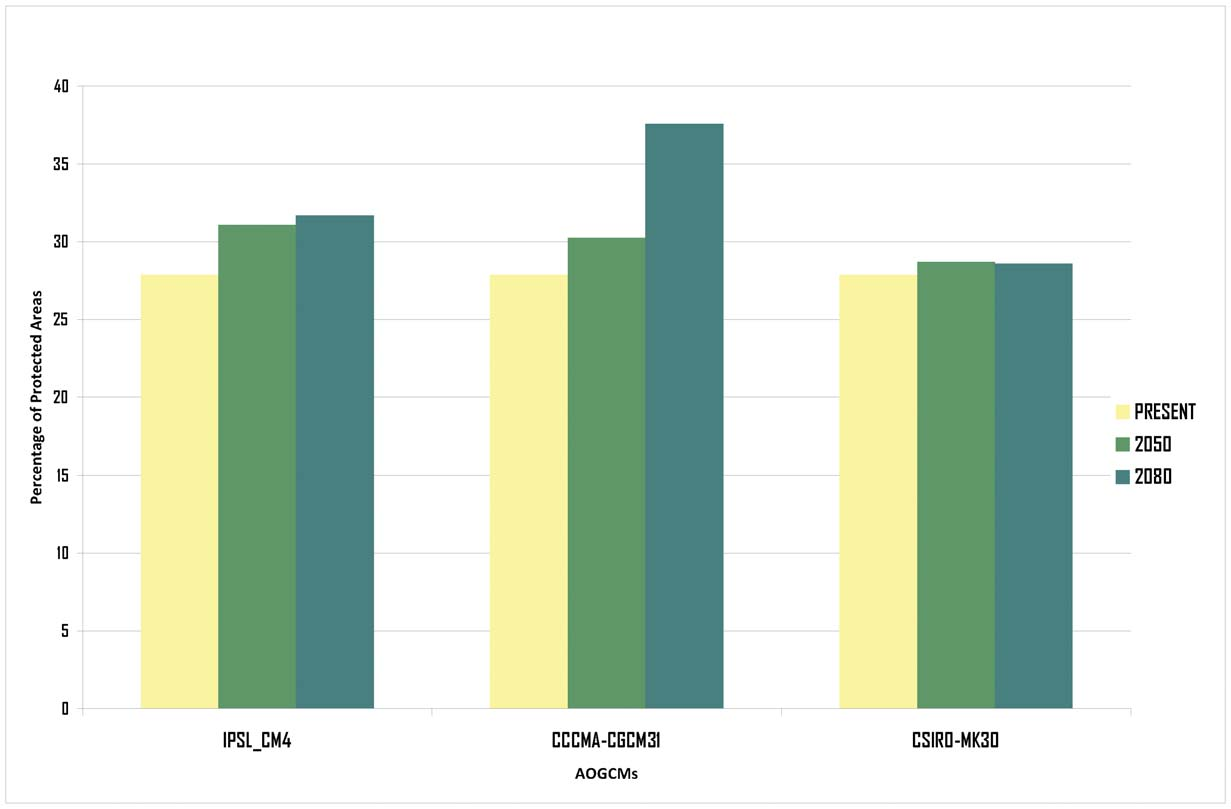
Histogram of suitable protected areas. Percentage of suitable surface of all the IUCN protected areas in the continent for *Lithobates catesbeianus* at the three analyzed time slices and AOGCMs.

The histogram in Fig. 5 shows the percentage of surfaces of the IUCN protected areas that are environmentally suitable for *L. catesbeianus*, which according to this analysis, would slightly increase towards the future.

## Discussion

### Current suitable areas for invasion

The southern portion of Brazil and northeastern Argentina (Atlantic Forest), central eastern Argentina and all of Uruguay (Fig. 1–2) show the highest values of suitability. Big portions of territories in these countries are also environmentally suitable for this alien species. This is in concordance with the fact that Brazil and Argentina are the countries with the most geographically extended biological invasion of the American Bullfrog in South America [24], [28]. It is not a coincidence, however, that they are the main producers of bullfrog culture in the continent [43]. Giovanelli et al. [23], Loyola et al. [27] and Nori et al. [24] mentioned that the presence of *L. catesbeianus* in this area is of special concern and here we provide new evidence supporting the vulnerability of this imperiled region.

Although reported populations in the northern and central western countries of the continent (Colombia, Peru, Ecuador and Venezuela) are located in suitable areas (Fig. 1–2), their suitability values were very low (Fig. 4), reflecting the great tolerance range of this alien species.

### Potential changes in the future

Although projections in different AOGCMs showed slight differences, all the scenarios can be characterized by the same pattern of change (see results of *I* and *RR*). In agreement with the hypothesis of Urbina-Cardona and Castro [25], we found a slight reduction in suitable surfaces for *L. catesbeainus* in South America towards the future (Fig. 1, 2, 3). However, areas that will continue to be highly suitable for this species are those where most populations have been reported and those that are of special concern in the continent (southern Brazil and northeastern of Argentina; Fig. 4) [23], [24], [27].

The greater retraction of suitable surfaces figures in the central portion of Brazil, however, more than 95% of alien populations in this country were reported in regions which, according to our analyses, will continue to be environmentally suitable [23]. On the other hand, the larger expansion of suitable surfaces for this invasive species is located in the northern portion of the continent, specifically in Colombia, northern Brazil, Ecuador and Peru (Fig. 1–2). We consider that this fact is of special concern because invasive populations of the bullfrog are currently restricted to reduced suitable surfaces, which means that an expansion would lead to an increase in their distributional range.

The Andean-Patagonian forest (southwestern Argentina and southeastern Chile), eastern Paraguay, and northwestern Bolivia have not been mentioned as a concern because invasive populations have not yet been found in those regions. But, according to our results, these areas would be optimal for the establishment of the species because they hold big extensions of suitable potential habitat for both current and futures conditions (see Fig. 1).

### Invasion of protected areas

Although we did find a slight reduction in suitable surfaces for *L. catesbeianus* towards the future, the surfaces of protected areas that are environmentally suitable for the species increased (Fig. 5). The reason for this result lies in the pattern of change of suitable surfaces; areas with the highest increase in suitable surfaces were those with a large percentage of IUCN protected areas, including most of Venezuela, western Colombia and Peru, and north central Brazil. In contrast, areas that showed the greatest retraction hold a considerably lower percentage of protected area surfaces. Additionally, our results showed considerable differences between different AOGCMs: while two of these climate models (IPSL_CM4 and CSIRO_MK30) reflected similar patterns of change (slight increase with respect to the present), the CCCMA-CGCM31 model showed a considerable increase in the last period (2080), probably because it included larger surfaces of reserves in southern Colombia and Venezuela and northern Brazil (Fig. 1).

We provide further evidence into what Loyola et al. [27] pinpointed: a retraction in suitable surfaces for *L. catesbeianus* in the western portion of Brazil could drive the alien species into protected areas currently established in the Atlantic Forest. Although our analysis did not show a robust pattern of change, we can assert that beyond a hypothetical retraction in potential at risk surface in the continent, this invasive alien species will continue to be an important threat to the network of protected areas established in South America.

### Methodological comments

Currently, SDMs are widely used to quantify the potential distribution of alien species [20]. These tools correlate environmental and topographic variables with observed distribution without taking into account physiological aspects of species, adopting the general assumption that the best indicator of a species' climatic requirements is its current distribution, and therefore resulting in estimations of the realized niche of the species [44], [45]. However, because invasive species in non-native areas are concern of several (and sometimes geographical independent) case studies [39], the application of this type of methodological protocol must take into account several important aspects for each particular case study.

Here we used the native range of the species for model calibration. Some studies have suggested that for the estimation of risk areas, models should be calibrated based on the “entire range” of the species (data of native plus invasive range) [3], [46]–[48]. Nevertheless, others authors have demonstrated that when using only data from the native range, one can make very accurate predictions of areas at risk [23]–[27], [39], [49], [50]. Particularly in alien amphibians, a recent paper pinpointed that invader establishment success is higher in areas with abiotic conditions similar to the native range [51]. In addition, the use of the distributional data from the invasive range of the species in model calibration implicitly makes an important assumption: all of the records used for model calibration represent viable populations (that survive and growth) that have colonized, established and are currently spreading along the landscape (*sensu* Hellmann et al. [52]). In this regard, alien populations are ecologically unknown and most of what is known has been published in the last five years [23], [24]. In practical terms, this means that we cannot assume the viability of the populations of the invaded range and the inclusion of these records for model calibration would probably bias our results. Finally, the great concordance between our results and invasive records of the species reported by field researchers are evidence that the selection of the calibration records was correct.

We also applied a minimum convex polygon to search for the novel conditions (Fig. S1). In contrast, Giovanelli et al. [23] and Nori et al. [24] used a large inset to calibrate their models, and generated a likely biased estimation of novel conditions, which could lead to mistakes in their final predictions. Further, we selected the “minimum training presence” value as a threshold for the model. In Giovanelli et al. [23] and Nori et al. [24], the authors used other threshold criteria and, as a consequence, they converted to null values a big portion of the at risk area. For example, in Giovanelli et al. [23], the major portion of the central east of Brazil (including a big part of the Cerrado and Atlantic Forest) appear converted to null values, even though invasive populations have been documented. Our analysis reflects that most of these sites, at current conditions, represent at risk areas (Fig. 1–2)**.**

### Management and policy recommendations

In order to control the spread of bullfrogs, the development of management policies should be based on sound science that characterizes the interactions between the species and climate change [53]. In this regard, SDMs are a cost-effective, early warning system that allows the identification of the most suitable areas of a potential invasion, thus giving the opportunity to prioritize and focalize actions as well as investments for certain regions. In order to control the spread of the existing *L. catesbeianus* populations, and to prevent further invasions in South America, we consider that the results of this study should be taken into account when identifying vulnerable areas and making management decisions.

Some management recommendations regarding the spread of *L. catesbeianus* in South America have been made in recent studies [23], [24], [26], [27]. We agree with the authors and consider that urgent measures should be taken in the Atlantic Forest. It is essential for governments to make additional efforts in collaborating with universities, research institutes, environmental government and non-government agencies, as well as environmental corporations. Continuous monitoring of the native biodiversity in this biome should be a priority since *L. catesbeianus* is likely to colonize reserves more efficiently under climate changes [27].

Prevention is the cheapest, most effective method for combating invasive species when compared with eradication or control [53]–[55]. Our results show that the Andean-Patagonian forest, eastern Paraguay and northwestern Bolivia, where *L. catesbeianus* has not yet been reported, are optimal places for the species to thrive. Therefore, we consider that importing, breeding and/or having individuals in captivity in these areas must be urgently forbidden and strictly regulated.

The most effective eradication programs could take place in Colombia, Venezuela, Ecuador and Peru, where the invasion of *L. catesbeianus* is restricted to “small areas” in poorly suitable environments. However, proper programs should begin in the near future because climate change could enable a considerable expansion of the species in these areas.

It is prioritary to generate a regional agenda to identify and isolate some wetlands and other natural ecosystems in which to conduct long-term monitoring of bullfrog populations and conduct experimental and ecological studies that allow us to better understand their behavior, reproductive biology, diet, competition with native species (at larval and adult stages), among other aspects. All of the above will allow the control of the dispersal of the species along permanent natural and artificial bodies of water such as irrigation districts for productive systems.

In the most vulnerable regions it is imperative to broad the population targets within the society so as to avoid the transport of bullfrogs used as pets or for food. Massive environmental campaigns must help local people identify the species, differentiate it from other native species, and be aware of the extreme damage that this species causes to ecosystem functions and services. Once the local people can identify the species and its preferred habitats, local government could begin an aggressive campaign to stimulate controlled hunting for bullfrog individuals which can be used as a source of food or in biomedical experiments in most (current and future) vulnerable regions.

This study provides more evidence highlighting the complexity of the *L. catesbeianus* problem in South America, as well as being useful for determining certain urgencies. However, we are aware that this type of research alone is not enough to resolve the problem. On one hand, a hard research line that answers some management-related questions is still needed [27]. On the other hand, a successful management to an imminent *L. catesbeianus* invasion in South America will only be possible if government agencies and related entities begin to play bigger role.

## Supporting Information

Figure S1 **Individual records of** ***Lithobares catesbeianus*** **from its native range used to perfom MaxEnt models and the minimum convex polygon used to calibrate the projections.**
(TIF)

Table S1 **Results of the Pearson correlation between the 20 variables.**
(XLS)
