# Supplementary figures and images for: Climate Change and American Bullfrog Invasion: What Could We Expect in South America?
Source: PLoS One. 2011 Oct 3;6(10):e25718. doi: 10.1371/journal.pone.0025718 (PMC3185029; doi:10.1371/journal.pone.0025718)

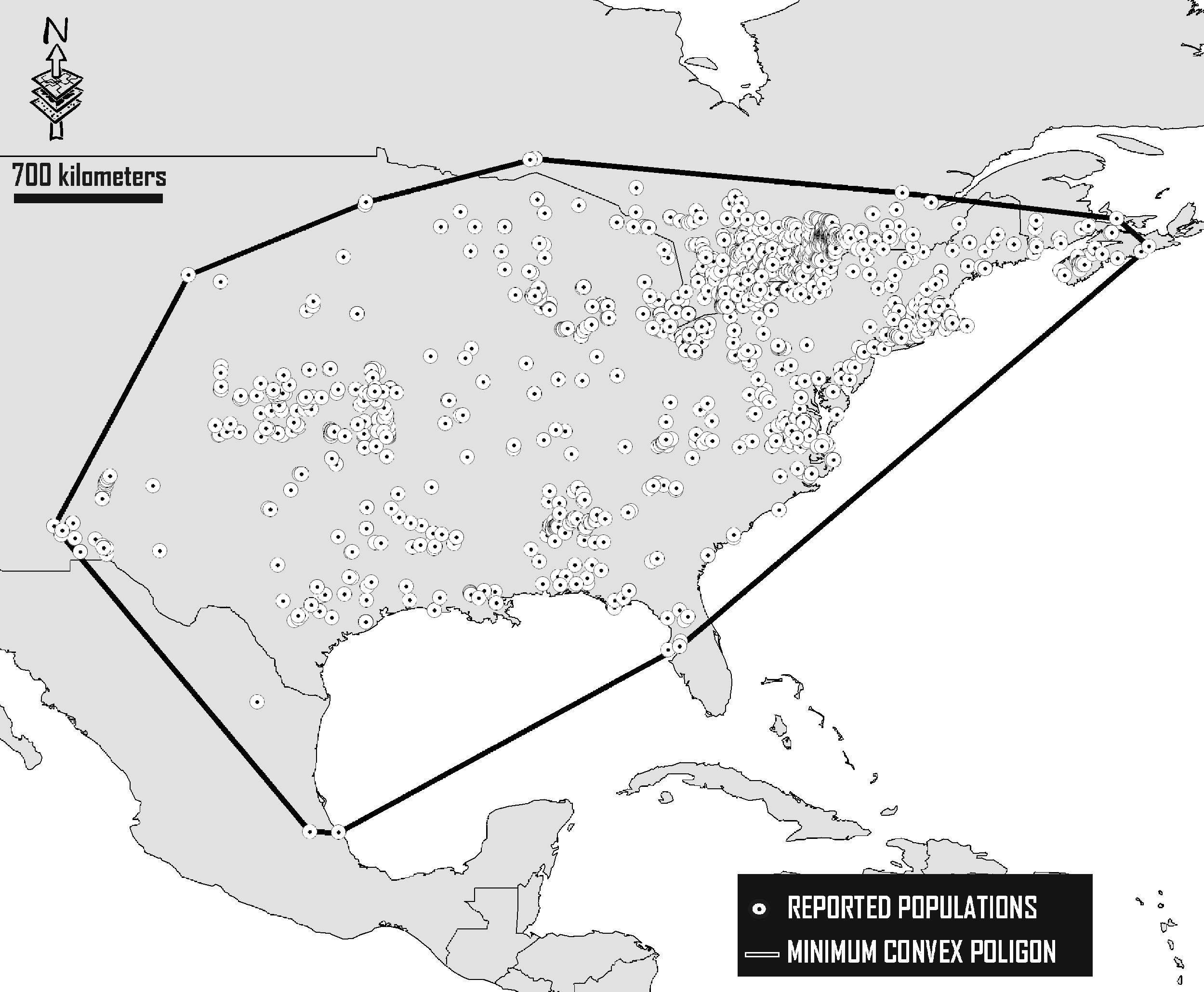

Supplement: Figure S1 — Individual records of Lithobares catesbeianus from its native range used to perfom MaxEnt models and the minimum convex polygon used to calibrate the projections. (TIF) [file pone.0025718.s001.tif]
